# Electrical Impedance Tomography for Cardio-Pulmonary Monitoring

**DOI:** 10.3390/jcm8081176

**Published:** 2019-08-07

**Authors:** Christian Putensen, Benjamin Hentze, Stefan Muenster, Thomas Muders

**Affiliations:** 1 Department of Anesthesiology and Intensive Care Medicine, University Hospital Bonn, 53127 Bonn, Germany; 2 Chair for Medical Information Technology, RWTH Aachen University, 52074 Aachen, Germany

**Keywords:** electrical impedance tomography, bioimpedance, image reconstruction, thorax, regional ventilation, regional perfusion, monitoring

## Abstract

Electrical impedance tomography (EIT) is a bedside monitoring tool that noninvasively visualizes local ventilation and arguably lung perfusion distribution. This article reviews and discusses both methodological and clinical aspects of thoracic EIT. Initially, investigators addressed the validation of EIT to measure regional ventilation. Current studies focus mainly on its clinical applications to quantify lung collapse, tidal recruitment, and lung overdistension to titrate positive end-expiratory pressure (PEEP) and tidal volume. In addition, EIT may help to detect pneumothorax. Recent studies evaluated EIT as a tool to measure regional lung perfusion. Indicator-free EIT measurements might be sufficient to continuously measure cardiac stroke volume. The use of a contrast agent such as saline might be required to assess regional lung perfusion. As a result, EIT-based monitoring of regional ventilation and lung perfusion may visualize local ventilation and perfusion matching, which can be helpful in the treatment of patients with acute respiratory distress syndrome (ARDS).

## 1. Introduction

Electrical impedance tomography (EIT) is a radiation-free functional imaging modality that allows non-invasive bedside monitoring of both regional lung ventilation and arguably perfusion. Commercially available EIT devices were introduced for clinical application of this technique, and thoracic EIT has been used safely in both adult and pediatric patients [1,2].

## 2. Basics of Bioimpedance

Bioimpedance can be defined as the voltage response of biological tissue to an externally applied alternating electric current (AC). It is commonly obtained using four electrodes, where two are used for AC injection and the other two for voltage measurement [3,4]. Thoracic EIT measures the regional distribution of intra-thoracic bioimpedance and can be seen as an extension of the four electrode principle to the image plane spanned by the electrode belt [1]. Dimensionally, electrical impedance (Z) is the same as resistance and the corresponding International System of Units (SI) unit is Ohm (Ω). It can be conveniently expressed as a complex number where the real part is resistance and the imaginary part is called reactance, which quantifies effects resulting from capacitance or inductance. Capacitance depends on the biomembranes’ characteristics of the tissue such as ion channels, fatty acids, and gap junctions, whereas resistance is mainly determined by the composition and the amount of extracellular fluid [1,2]. At frequencies below 5 kilohertz (kHz), electrical current flows through extracellular fluid and is primarily dependent on the resistive characteristics of the tissues. At higher frequencies up to 50 kHz, electrical currents are slightly deflected at cell membranes which leads to an increase of capacitive tissue properties. At frequencies above 100 kHz, electrical current can pass through cell membranes and decrease the capacitive component [1,2]. Therefore, the effects that determine tissue impedance strongly depend on the utilized stimulation frequency. Bioimpedance is usually given as resistivity or conductivity, which normalize resistance or conductance to unit area and length. The corresponding SI units are Ohm-meter (Ω·m) for resistivity and Siemens per meter (S/m) for conductivity. Resistivity of thoracic tissue ranges from 150 Ω·cm for blood, to 700 Ω·cm for deflated lung tissue, up to 2400 Ω·cm for inflated lung tissue (Table 1). In general, tissue resistivity or conductivity depends on the fluid content and ion concentration. In terms of the lungs, it also depends on the amount of air in the alveoli. While most tissues show isotropic behavior, heart and skeletal muscle behave anisotropic, meaning that resistivity strongly depends on the direction in which it is measured.

## 3. EIT Measurements and Image Reconstruction

To perform EIT measurements, electrodes are placed around the thorax in a transverse plane, usually in the 4th to 5th intercostal spaces (ICS) at the parasternal line [5]. Subsequently, the changes of impedance can be measured in the lower lobes of the right and left lungs, as well as in the heart region [1,2]. To place the electrodes below the 6th ICS might be difficult as the diaphragm and abdominal content periodically enter the measurement plane.

Electrodes are either single self-adhesive electrodes (e.g., electrocardiogram, ECG) that are placed individually with equal spacing in-between the electrodes or are integrated in electrode belts [1,2]. Also, self-adhesive stripes are available for a more user-friendly application [1,2]. Chest wounds, chest tubes, non-conductive bandages or conductive wire sutures may preclude or significantly affect EIT measurements. Commercially available EIT devices usually use 16 electrodes, but EIT systems with 8 or 32 electrodes are also available (please see Table 2 for details) [1,2].

**Figure 1:**
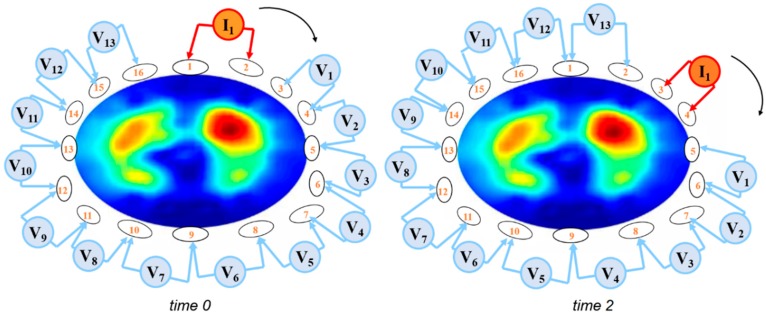
Current application and voltage measurements around the thorax using an EIT system with 16 electrodes. Within a few milliseconds, both the current electrodes and the active voltage electrodes are repeatedly rotated around the thorax.

During an EIT measurement sequence, small AC (e.g., <5 mA at a frequency of 100 kHz) are applied through different pairs of electrodes and the resulting voltages are measured using the remaining other electrodes [6]. Bioelectrical impedance between the injecting and the measuring electrode pairs is calculated from the known applied current and the measured voltages. Most commonly, adjacent electrode pairs are used for AC application in a 16-elektrode system, while 32-elektrode systems often apply a skip pattern (see Table 2) to increase the distance between the current injecting electrodes. The resulting voltages are measured using the remaining electrodes. Currently, there is an ongoing discussion about different current stimulation patterns and their particular advantages and disadvantages [7]. To obtain a full EIT data set of bioelectrical measurements, the injecting and the measuring electrode pairs are continuously rotated around the entire thorax (Figure 1).

The AC used during the EIT measurements are safe for a body surface application and remain undetected by the individual patient. For safety reasons, the use of EIT in patients with electrically active devices (e.g., cardiac pacemakers or cardioverter-defibrillators) is not recommended.

The EIT data set which is recorded during one cycle of AC applications is technically termed a frame and contains the voltage measurements to generate the raw EIT image. The term frame rate reflects the number of EIT frames recorded per second. Frame rates of at least 10 images/s are required to monitor ventilation and 25 images/s to monitor cardiac function or perfusion. Commercially available EIT devices use frame rates between 40 and 50 images/s [2], as shown in Table 2.

To generate EIT images from the recorded frames, the so-called image reconstruction is applied. Reconstruction algorithms aim to solve the inverse problem of EIT, which is the recovery of the conductivity distribution inside the thorax based on the voltage measurements that have been acquired at the electrodes on the thorax surface. Initially, EIT reconstruction assumed that electrodes were placed on a circular or ellipsoid plane, whereas newer algorithms utilize information about the anatomical shape of the thorax. Currently, the Sheffield back-projection algorithm [8], the finite element method (FEM) based linearized Newton-Raphson algorithm [9], and the Graz consensus reconstruction algorithm for EIT (GREIT) [10] are frequently used.

In general, EIT images are comparable to a two-dimensional computed tomography (CT) image: these images are conventionally rendered so that the operator looks from caudal to cranial when analyzing the picture. In contrast to a CT image, an EIT image does not display a “slice” but an “EIT sensitivity region” [11]. The EIT sensitivity region is a lens-shaped intra-thoracic volume from which impedance changes contribute to the EIT image generation [11]. Shape and thickness of the EIT sensitivity region depend on the dimensions, the bioelectric properties, and the shape of the thorax as well as on the utilized current injection and voltage measurement pattern [12].

**Figure 2:**
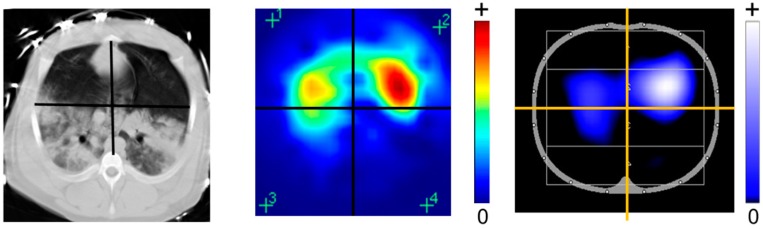
Different available color codings of EIT images in comparison to the CT scan. The rainbow-color scheme uses red for the highest relative impedance (e.g., during inspiration), green for a medium relative impedance, and blue for the lowest relative impedance (e.g., during expiration). A newer color scales use instead black for no impedance change), blue for an intermediate impedance change, and white for the strongest impedance change.

Time-difference imaging is a technique that is used for EIT reconstruction to display changes in conductivity rather than the absolute conductivity levels. A time-difference EIT image compares the change in impedance to a baseline frame. This affords the opportunity to trace time-varying physiological phenomena such as lung ventilation and perfusion [2]. Color coding of EIT images is not unified but commonly displays the change in impedance to a reference level (Figure 2). EIT images are usually coded using a rainbow-color scheme with red indicating the highest relative impedance (e.g., during inspiration), green a medium relative impedance, and blue the lowest relative impedance (e.g., during expiration). For clinical applications, an interesting option is to use color scales ranging from black (no impedance change) to blue (intermediate impedance change), and white (strong impedance change) to code ventilation or from black, to red, and white to mirror perfusion.

## 4. Functional Imaging and EIT Waveform Analysis

**Figure 3:**
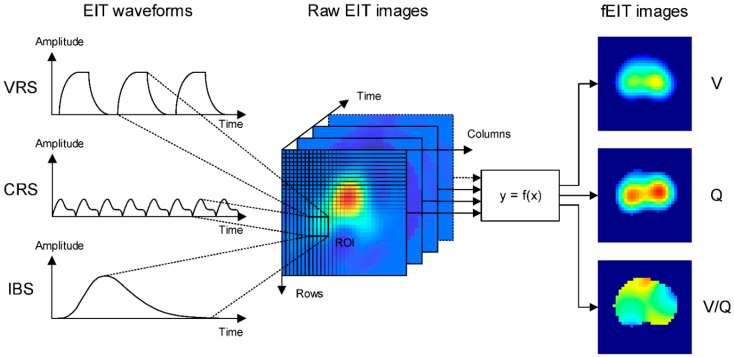
EIT waveforms and functional EIT (fEIT) images are derived from the raw EIT images. EIT waveforms can be defined pixel-wise or on a region of interest (ROI). Conductivity changes result naturally from ventilation (VRS) or cardiac activity (CRS) but can also be induced artificially, e.g., by bolus injection (IBS) for perfusion measurement. fEIT images display regional physiological parameters, such as ventilation (V) and perfusion (Q), extracted from the raw EIT images using a mathematical operation over time.

Analysis of EIT data is based on EIT waveforms that are formed in individual image pixels in a series of raw EIT images over time (Figure 3). A region of interest (ROI) can be defined to summarize activity in individual pixels of the image. In each ROI, the waveform displays changes in regional conductivity over time resulting from ventilation (ventilation-related signal, VRS) or cardiac activity (cardiac-related signal, CRS). Additionally, electrically conductive contrast-agents such as hypertonic saline can be used to obtain an EIT waveform (indicator-based signal, IBS) and may be linked to lung perfusion. The CRS may originate from both the cardiac and lung region and may be partly attributed to lung perfusion. Its exact origin and composition are incompletely understood [13]. Frequency spectrum analysis is frequently used to discriminate between ventilation- and cardiac-related impedance changes. Non-periodic changes in impedance may be caused by changes in ventilator settings.

Functional EIT (fEIT) images are generated by applying a mathematical operation on a sequence of raw images and the corresponding pixel EIT waveforms [14]. Since the mathematical operation is applied to calculate a physiologically relevant parameter for each pixel, regional physiological characteristics such as regional ventilation (V), respiratory system compliance as well as regional perfusion (Q) can be measured and displayed (Figure 3). Data from EIT waveforms and simultaneously registered airway pressure values can be utilized to calculate the lung compliance as well as lung opening and closing for each pixel using changes of pressure and impedance (volume). Comparable EIT measurements during stepwise inflation and deflation of the lungs allow the displaying of pressure-volume curves on a pixel level. Depending on the mathematical operation different types of fEIT images may address different functional characteristics of the cardio-pulmonary system.

## 5. Ventilation Monitoring

### 5.1. Validation of EIT Measurements

#### 5.1.1. Global Ventilation

Global ventilation has been shown to correlate with global impedance changes (∆Z_global_) which result from sequential EIT measurements in healthy or injured lungs by using a super-syringe technique [15], plethysmography [16], or spirometry [17]. Tidal impedance variation (TIV) represents ∆Z which is generated during a tidal breath and equals the difference between the maximum and minimum impedance at end-inspiration and end-expiration. Global TIV can be scaled and converted to volume (mL) with sufficient accuracy using the measurements of tidal volume (V_T_).

#### 5.1.2. Global Changes in End-Expiratory Lung Volume (EELV) and Impedance (EELI)

Changes in EELV (∆EELV) and EELI (∆EELI) were investigated during lung inflation using PEEP-trials or recruitment maneuvers in both healthy and injured lungs. Global ∆EELI has been found to correlate (*R*^2^ = 0.92–0.95) with global ∆EELV which has been determined by spirometric measurements of V_T_ [17,18], multi-breath nitrogen-washout technique [19], CT [20], and electron-beam computed tomography (EBCT) [21]. Data from patients with acute respiratory distress syndrome (ARDS) indicate a decrease in the linear relationship between global ∆EELI and ∆EELV, which may be caused by a heterogeneous lung injury [19]. For clinical purposes, ∆EELI can be scaled and converted to volume (mL) with sufficient accuracy by using the global tidal volume variation which results from a change in PEEP.

#### 5.1.3. Regional Changes in Lung Ventilation or Volume

Regional ventilation which has been assessed by regional impedance changes (∆Z_regional_) using EIT correlate with data derived by EBCT (*R*^2^ = 0.81–0.93). In ARDS patients, an excellent correlation (*R*^2^ = 0.96) was observed between ∆Z_regional_ and regional air content changes determined by CT [20], dynamic X-ray CT [20], single-photon emission computed tomography (SPECT) [22], and positron emission tomography (PET) [21].

These data suggest that changes in relative impedance measured by EIT can be used to quantify regional ventilation and lung volumes.

### 5.2. Analyzing Spatial Distribution of Ventilation

#### 5.2.1. Subtracting fEIT Images

**Figure 4:**
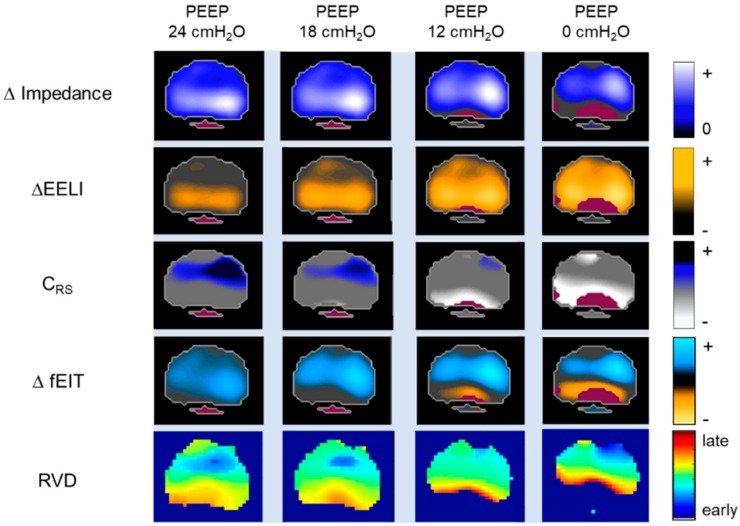
Change in impedance (∆Impedance), change in end-expiratory lung impedance (∆EELI), regional respiratory system compliance (C_RS_), difference functional EIT (∆fEIT) images, and regional ventilation delay (RVD) are determined at different PEEP-levels. The ∆EELI images display the regional ∆EELI between the different positive end-expiratory pressure (PEEP) levels. Regional C_RS_ is measured and visualized in fEIT image by dividing pixel-by-pixel tidal impedance variations by global driving pressure. The ΔfEIT images are created by subtracting fEIT before the PEEP step from fEIT after each PEEP step. The RVD shows regional temporal ventilation delay and thus the temporal ventilation heterogeneity caused by a change in PEEP.

Changes in lung function can be quantified by pixel-by-pixel subtraction of an fEIT image from a previous fEIT image. To obtain an approximation for changes in regional V_T_, the difference is normalized to the V_T_ divided by ∆Z_global_ (Figure 4).

The subtraction of fEIT images visualizes and measures the regional gain or loss of ventilation and lung volume which is caused either by changes of the ventilator settings (e.g., PEEP) or maneuvers (e.g., recruitment maneuvers, prone positioning). Thus, the substration of fEIT images localizes and quantifies recruitment and derecruitment of the lungs (Figure 4).

#### 5.2.2. Impedance Ratio

The impedance ratio divides the ventilation activity of the ventral region by the dorsal ventilation activity of the fEIT images, and may indicate both lung recruitment and derecruitment [23].

#### 5.2.3. Regional Respiratory System Compliance (C_RS_)

The C_RS_ during mechanical ventilation can be calculated when the V_T_ is divided by the driving pressure. When the EIT with simultaneously registered airway pressures is used, global and regional C_RS_ can be measured and visualized in fEIT images by dividing the pixel-by-pixel TIV by the global driving pressure (Figure 4) [24,25]. Regional C_RS_ has been suggested to localize and quantify the regional alveolar collapse and overdistension during ventilation [24,25].

#### 5.2.4. Regional Pressure–Volume (P/V) Curves

Global quasi-static P/V curves have been recorded during low-flow inspiratory and expiratory maneuvers to identify the airway pressure which is required to open collapsed (lower inflection point, LIP) and overdistended lung units (upper inflection point, UIP). Regional quasi-static P/V curves can be generated using ∆Z in a pixel or ROI when the global airway pressure has been registered simultaneously. Regional LIP and UIP vary and reflect heterogeneity in lung mechanics [26]. The resulting regional opening pressures (LIP) vary and are highest in the dorsal lung regions. Using fEIT images to visualize the regional distribution of LIP and UIP in different lung regions might be a helpful tool to individually titrate PEEP [27,28]. The shape of regional P/V curves can be quantified and can be displayed in a color-coded map to visualize regional tidal recruitment and overdistension [29].

#### 5.2.5. Alveolar Overdistension and Collapse (ODCL)

ODCL is based on the pixel-by-pixel monitoring of C_RS_ during a PEEP trial with a fixed driving pressure [30]. This maneuver identifies the highest compliance and the best pixel C_RS_ for a specific PEEP-level. Lung collapse is indicated when the reduction of the PEEP results in a lower pixel C_RS_. Lung overdistension is assumed when an increased PEEP results in a lower pixel C_RS_ [30]. The percentage of lung collapse or overdistension can be calculated in a pixel-by-pixel manner by subtracting a C_RS_ at a given PEEP level from the best C_RS_ and referencing it to the best C_RS_. Accumulated collapse/overdistension for the entire lung at each PEEP step is calculated as a weighted average of all collapsed/overdistended pixels, where the weighting factor is the best pixel compliance [30]. In an fEIT image, ODCL displays and quantifies lung collapse/overdistension at the pixel level and can be helpful to titrate PEEP and to prevent both alveolar collapse and overdistension. Recently, ODCL-measurements were used to demonstrate that a decremental PEEP titration preceded by a recruitment maneuver obtained the best lung function by decreasing both collapse and overdistension in severely obese ARDS patients [31].

#### 5.2.6. Center of Ventilation (CoV)

The center of ventilation (CoV) is a measure to describe the spatial distribution of pulmonary ventilation. It was initially defined as the weighted mean of the geometrical centers of ventilation of the left and right lung in the dorsal-ventral direction [32]. A CoV of 50% indicates a ventilation distribution that is centered in orsal-ventral direction of the thorax. Reduced values of the CoV indicate a shift of ventilation distribution towards ventral lung regions, which may be observed during derecruitment due to alveolar collapse in the dependent lung regions [32]. However optimum values of the CoV highly depend on chest anatomy of the individual patient. Following the definition of the center of gravity (CoG) in mechanics, more recent studies directly calculate a measure similar to the CoV in both dorsal-ventral [33] and right-left direction [34] as the weighted means of the image row or column sums, respectively. A detailed comparison of CoV and CoG can be found in reference [35]. Even though both measures behave very similarly, care should be taken when evaluating and comparing studies, as the definitions are not directly interchangeable.

In patients at risk for postoperative pulmonary complications, CoV was used to show that ventral redistribution of ventilation persisted up to the third postoperative day after abdominal surgery, whereas only minor effects were observed after peripheral surgery [36].

#### 5.2.7. Global Inhomogeneity Index (GI Index)

The GI index was introduced to quantify V_T_ distribution within the lungs and aims to summarize the complex pulmonary impedance distribution pattern using a single numeric value [37,38]. Calculation of the GI index and modifications to adapt to low-flow inflation have been described [37] and requires EIT images at two selected time points to calculate the median impedance value within an ROI. The differences in impedance variation between each pixel and the median value of all pixels are calculated and normalized to the sum of impedance values, in order to make the GI index universal and inter-patient comparable [37]. The GI index, used to titrate PEEP, investigate changes in ventilation distribution, and the progression of obstructive lung disease [37]. During a PEEP-trial, increase in PEEP resulted in a parabolic curve of the GI index. The PEEP level at which the GI value was minimal corresponded to the highest global dynamic compliance and lowest ventilator inhomogeneity and was suggested to indicate the optimal PEEP level [37].

#### 5.2.8. Dependent (DSS) and Non-Dependent Silent Spaces (NSS)

Silent spaces have been defined as poorly ventilated lung regions which show impedance changes of less than 10% of the maximal end-expiratory to end-inspiratory impedance change on lung ROIs. These ROIs were derived from CT scans that were mapped onto the EIT images [39]. The silent spaces can be separated into dependent silent spaces (DSS) and non-dependent silent spaces (NSS) based on a horizontal line which passes through the CoV [39]. NSS might be indicative for overdistension [39], whereas changes in DSS have been shown to correlate with changes in recruited and derecruited lung volume, as measured by the global P-V curve, during incremental and decremental PEEP steps [40].

### 5.3. Analyzing Temporal Distribution of Ventilation

#### 5.3.1. Regional Ventilation Delay (RVD)-Regional Ventilation Delay Inhomogeneity (RVDI)

In an inhomogeneous lung with atelectasis formation, higher airway pressure is required to open and ventilate the collapsed lung regions. Thus, the ventilation of these initially collapsed and recruited alveolar regions is delayed. The high temporal resolution of EIT allows the analysis of aeration and ventilation time courses. The regional ventilation delay (RVD) is an EIT measure that can show a temporal delay in regions of the lung and therefore the temporal heterogeneity occurring in the ventilated lung by the relationship between ∆Z and the ventilation time course in each pixel [20,41]. The RVD is the delay time needed for a regional impedance-time curve to reach a certain threshold of its maximal impedance change, which is normally set between 30 to 40% of its maximal impedance value during low-flow inflation [20,41]. RVDI is the standard deviation of RVD in all pixels [20,41] and quantifies temporal heterogeneity of regional ventilatory time courses. A small RVDI indicates a more homogeneous, a high RVDI an inhomogeneous regional ventilation delay distribution. In an fEIT image, RVD pixel values can be localized and quantified by color coding (Figure 4). RVDI was also calculated from regular breaths during mechanical ventilation, assisted, and spontaneous breathing efforts. However, previous research [41] demonstrated that a slow inflation maneuver is mandatory for sufficient RVDI calculation and assessment of tidal recruitment. Recently, it was demonstrated that the low inflation tidal volume during RVDI measurements can be reduced to 6–9 mL/kgBW [42]. This significantly reduces end-inspiratory pressures during slow inflation and further improves the clinical applicability of this method.

#### 5.3.2. Intratidal Gas Distribution-Intratidal Ventilation Index (ITVI)

ITVI analyses the ventilation homogeneity during the inspiration [43] and equals the amount of the impedance distributed to a predefined ROI within one inspiration. ITVI is calculated by dividing the inspiratory part of the global impedance curve into eight equal volume sections (12.5% of the entire inspiration). Then, sequential corresponding time points of the eight iso-volume steps are translated to the regional ITVI curves. ITVI gives the percentile contribution of the inspired gas distributed to the selected lung region at a certain time point. ITVI could demonstrate that during spontaneous breathing, ventilation is distributed more to the dorsal region, resulting from the diaphragm contraction in the early phase of inspiration.

#### 5.3.3. Regional Expiratory Time Constants

Whereas RVDI and ITVI describe the temporal behavior of different lung regions during inspiration, regional expiratory time constants can be used to quantify regional heterogeneity during expiration. Time constants are usually obtained from the global pneumatic volume curve. By definition, the time constant τ represents the time for V(t) to exhale 2/3 of its volume, whereas 3τ defines the time to reach at least 95% of complete exhalation. Values of τ can also be calculated per EIT image pixel on a breath by breath basis [44]. Data from patients with severe respiratory failure indicate that the distribution pattern of regional τ values depends on the type of respiratory failure [44]. Short regional τ values showing a narrow homogeneous frequency distribution were found in patients with pneumonia and restrictive lung diseases. In contrast, COPD patients showed a widespread inhomogeneous distribution of long lasting regional τ values, which was influenced by the PEEP level. These data suggest that regional τ might be related to expiratory flow limitation and may be useful to individually optimize PEEP level and ventilation strategy in different lung conditions [44].

### 5.4. Clinical Application

Regional measures derived from an EIT allow to localize and quantify ventilation heterogeneity under dynamic conditions. Thus, EIT has the potential to monitor heterogeneities in regional ventilation or guide mechanical ventilation in patients with ARDS [45] or chronic obstructive lung disease [46].

#### 5.4.1. Estimation of Lung Volume, Collapse and Overdistension

Continuous registration of ∆Z_global_ and ∆Z_regional_ or EIT derived parameters, such as regional C_RS_, regional P/V curves, GI index ODCL, RVDI, and ITV can be helpful to maximize the lung recruitment and to reduce alveolar overdistension using recruiting maneuvers, PEEP-trials, or V_T_ adjustment.

Bikker et al. [47] observed during a decremental PEEP trial in patients with and without ARDS, that ventilation distribution change maps (ΔfEIT maps) can visualize gain or loss of regional ventilation in dependent and non-dependent lung areas at the bedside. ΔfEIT and regional C_RS_ maps during a stepwise decrease in PEEP were significantly different between patients with and without ARDS, indicating a different PEEP dependency between these two groups and between individual patients. By using a recruitment maneuver and a decremental PEEP trial, Lowhagen et al. [43] demonstrated that ITV and ΔEELI offer additional information on recruitability and optimal PEEP in patients with ARDS. Zick et al. [48] showed that ventilation distribution, regional C_RS_, and ∆EELI can be used to assess tidal recruitment and end-inspiratory overinflation in patients with ARDS. In patients with and without ARDS, Zhao et al. [38] demonstrated that the GI index is a reliable measure of ventilation heterogeneity and highly correlates with recruitment determined using EIT. Cinnella et al. [34] observed in patients with ARDS have a decrease in the ROIventral/dorsal impedance tidal variation ratio following a lung recruitment using an open lung ventilator strategy. Decrease in the ROIventral/dorsal impedance tidal variation ratio was associated with a decrease in lung elastance and driving pressure [34]. Thus, the decrease in ROI ventral/dorsal impedance tidal variation ratio which was induced by the lung recruitment maneuver was interpreted as a clear sign of lung recruitment, which occurred predominantly in the dorsal lung regions [34]. Yun et al. [49] suggested that EIT has the potential to evaluate if ARDS patients respond to a recruitment. Mauri et al. [50] showed an improved homogeneous ventral-to-dorsal ventilation distribution during assisted ventilation at a high PEEP-level when compared with control mechanical ventilation. Zhao et al. [51] compared individualized PEEP levels which were obtained from ODLC measurements to PEEP levels which were derived from global P/V curves (PEEP 2 cmH_2_O above LIP) and demonstrated that EIT-based PEEP titration might improve lung mechanics, gas exchange, and weaning success. ODLC measurements were also used to individually titrate PEEP in ARDS patients during ultra-protective mechanical ventilation (using a V_T_ of 4 mL/kg BW or below) who underwent extra corporeal membrane oxygenation [52]. This study showed a heterogeneous distribution of individual PEEP levels providing the best compromise between collapsed and overdistended lung. Mauri et al. [53] studied the effect of ventilatory support using a high-flow nasal cannula (HFNC) in hypoxemic respiratory failure and demonstrated that increasing the HFNC flow rate decreased inspiratory effort and increases end-expiratory lung volume as measured by ΔEELI.

#### 5.4.2. PEEP Titration in Obese Patients

Nestler et al. found that a PEEP strategy which minimizes RVDI indicating an increase in temporal heterogeneity of the lung during a decremental PEEP trial improved gas exchange, lung mechanics, and ventilation distribution in severely obese patients who underwent laparoscopic surgery [54]. Recently, ODCL-measurements were used by Fumagalli et al. to demonstrate that a decremental PEEP titration preceded by a recruitment maneuver obtained the best lung function by decreasing both collapse and overdistension in severely obese ARDS patients [31].

#### 5.4.3. Pneumothorax Detection

EIT has been shown to reliably detect the development of pneumothorax at the bedside in real-time [55]. Initially, the EIT scan displays a clear rapid increase in regional impedance with an associated decrease in ΔEELI at the side of the pneumothorax. The change in aeration images showed a similar clear increase. About 30 seconds after onset, an increase in EELI is observed at the side of the pneumothorax, which is explained by ongoing increasing pleural gas accumulation [56]. EIT has been shown to detect a pneumothorax within three ventilator cycles with 100% sensitivity [56] and to be useful for early bedside detection of pneumothorax in patients with ARDS [57].

#### 5.4.4. Detection of Pleural Effusion

Impedance changes can paradoxically decrease during inspiration. These “out of phase impedance changes” have been attributed to pleural effusion by Becher and co-workers [58]. In this study (which was performed in 20 ARDS patients), the sum of out of phase impedance changes was increased when compared to the control patients and was decreased after pleural effusion drainage.

#### 5.4.5. Prediction of Weaning Success

Regional lung volume and ventilation distributions were monitored using EIT in 15 patients during spontaneous breathing trials by Zhao and co-workers [59]. It was demonstrated that redistribution of ventilation towards non-dependent lung regions after decreasing ventilatory support was associated with higher weaning success. These data suggest that the EIT-based measurement of intra-tidal gas distribution might be helpful to monitor weaning after prolonged mechanical ventilation.

#### 5.4.6. Monitoring Lung Volumes during Endotracheal Suctioning

EIT has been shown to be helpful for localizing and quantifying the loss of lung volume caused by open endotracheal suctioning and to titrate recruitment maneuvers to restore ventilation distribution [60].

#### 5.4.7. Monitoring Positioning of Endotracheal Tubes

Asymmetrical distribution of ventilation measured by EIT allows early detection of one-lung ventilation caused by inadvertent malpositioning of the endo-tracheal tube [61].

#### 5.4.8. Monitoring Ventilatory Dyssynchrony

During assisted spontaneous breathing, EIT may assist in early identification of potentially harmful asynchronies, such as breath stacking and pendelluft [62,63].

Breath stacking may be caused by reverse triggering or double-triggering and results in consecutive inspiratory cycles delivered by the ventilator on top of an incomplete exhalation [62]. During breath stacking, EIT detects potential harmful inspired end-inspiratory lung volumes even if regular monitoring only indicates a moderate increase in V_T_.

Pendelluft describes an intrapulmonary asynchrony caused by gas movement between different pulmonary regions resulting in alveolar de-inflation and inflation. EIT allows for the monitoring of a pendelluft phenomenon due to a movement of gas within the lung from nondependent to dependent regions without change in V_T_ [63]. This phenomenon can be caused by intense diaphragmatic contraction during excessive spontaneous efforts and has been suggested to cause unsuspected overstretch of dependent lungs during early inflation. Pendelluft can only be visualized by EIT but not by conventional monitoring of flow, volume, or pressure curves [63].

## 6. Perfusion Monitoring

Intermittent and invasive monitoring is based on EIT waveforms which result from contrast-agents (IBS) that are injected through a central venous catheter (CVC). Continuous and noninvasive monitoring can be performed based on cardiac activity (CRS) in both the heart and the lung region.

### 6.1. Perfusion Monitoring Using Contrast Agents

#### 6.1.1. Measurement Principle

**Figure 5:**
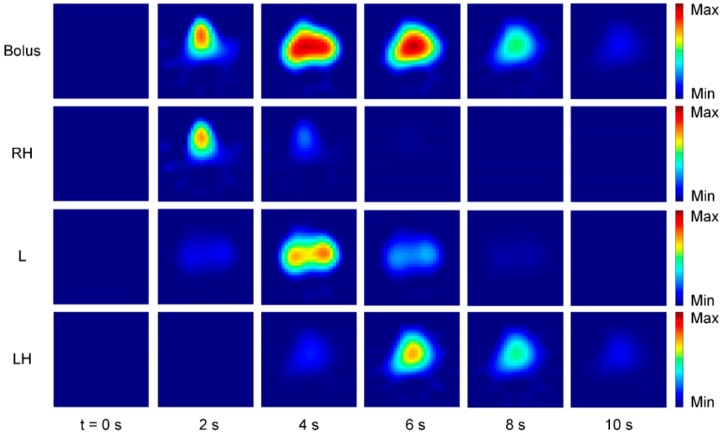
Bolus injection and transport of contrast agent (NaCl 10%) through the cardio-pulmonary system. The bolus is injected at *t* = 0 s and transported from the right heart (RH) to the lung (L) into the left heart (LH). Separation of the bolus is achieved using gamma-variate model fitting.

A promising technique for EIT perfusion monitoring is the use of conductive contrast agents such as hypertonic saline (e.g., NaCl 5.85%, 10 mL) to obtain an EIT waveform (indicator-based signal, IBS). The contrast agent is injected into the bloodstream as a bolus through a CVC under short-time apnea [64,65] and follows the blood flow through the cardio-pulmonary system (Figure 5).

Since the conductivity of the bolus differs from blood, it leaves a trace of impedance change wherever it passes by. Regional lung perfusion can then be extracted from the IBS by gamma-variate model fitting [64]. The IBS can be separated into disjoint image streams which correspond to the activity in the right heart (RH), the lung (L) and the left heart (LH) (Figure 5). While the IBS has the advantage of being clearly linked to regional perfusion, disadvantages might result from the need for a CVC, short-time apnea as well as possible hemolysis due to the high osmolality of the contrast agents [66]. Currently, the method is established in animal research, but a reduction of the contrast agent concentration might enable a clinical application in patients in the near future [65,67].

#### 6.1.2. Validation of Regional Perfusion

Brown et al. initially introduced the use of EIT with contrast agents for perfusion imaging [68]. Here, a bolus of isotonic saline (10 mL) was injected into a cubital vein [68]. Afterward, Frerichs et al. [69] showed a feasibility study for lung perfusion imaging with EIT using hypertonic saline (NaCl 5.85%, 10 mL) in comparison with electron-beam computed tomography (EBCT). Borges et al. [64] found a correlation of lung perfusion from EIT with SPECT using hypertonic saline (NaCl 20%, 10 mL) in healthy subjects with unilateral lung collapse and injured subjects with bilateral-dependent lung collapse. Reinius et al. [70] investigated regional ventilation and perfusion with EIT using hypertonic saline (NaCl 20%, 5 mL) during one-lung ventilation (OLV) at two different PEEP levels (5 and 10 cmH_2_O). The authors concluded that independent left and right-sided monitoring of lung ventilation and perfusion with EIT is a feasible method and might improve perioperative management during sequential OLV with capnothorax. A limitation of the study is that the investigators did not compare their findings to a valid reference method. Recently, Bluth et al. [67] compared lung perfusion derived by EIT with PET as a reference method using three saline concentrations (NaCl 10%, 5%, and 3%, 10 mL) during different conditions such as OLV, atelectasis, pulmonary artery occlusion and at two different PEEP levels. The authors found a correlation of EIT and PET under all conditions and concluded that indicator based EIT with 3% saline may prove useful for the assessment of regional pulmonary function at the bedside.

#### 6.1.3. Regional Ventilation to Perfusion Ratio (V/Q)

**Figure 6:**
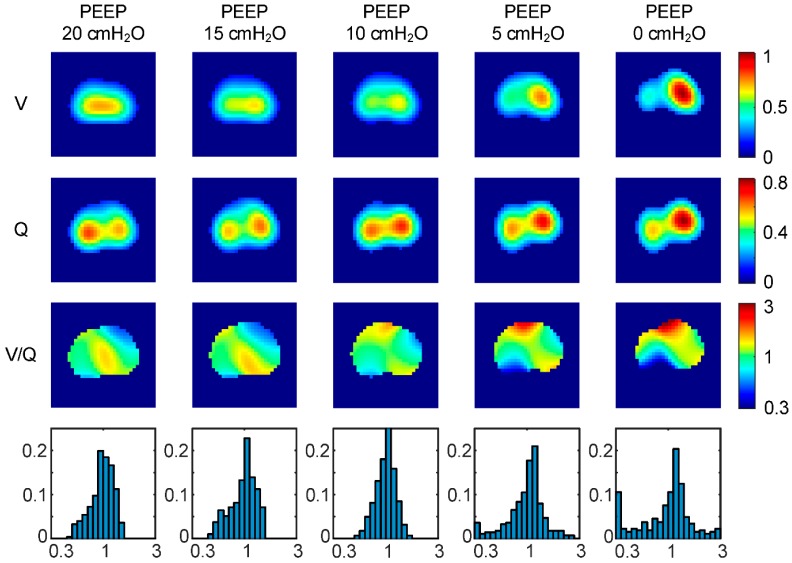
Regional lung ventilation (V) and perfusion (Q) obtained by EIT using a contrast agent in a decremental PEEP trial. Homogeneity of the distribution of regional ventilation to perfusion ratio (V/Q) slightly increases from 20 to 10 cm H_2_O, whereas a strong homogeneity decrease is observed from 10 to 0 cm H_2_O.

For the assessment of pulmonary gas exchange, it would be interesting to obtain regional ventilation to perfusion ratio (V/Q) at the bedside. To analyze the feasibility of the aforementioned method, we extracted both regional ventilation and perfusion from EIT measurements with hypertonic saline (NaCl 10%, 10 mL) during a decremental PEEP trial [71]. Subsequently, functional images of V/Q and corresponding histograms were derived by calculating the pixel-wise quotient of the mean normalized ventilation (V) and perfusion (Q) images (Figure 6).

We observed a slight increase in homogeneity of the distribution of regional V/Q from PEEP 20 to 10 cmH_2_O, whereas a strong decrease resulted from 10 to 0 cmH_2_O. These findings might imply that there is a zone of optimum PEEP which is likely to result in optimum gas exchange with respect to the distribution of regional V/Q. Furthermore, an increased number of pixels at the lower and upper ends of the V/Q range might indicate pathological phenomena, such as venous admixture or shunt perfusion at PEEP 5 and 0 cmH_2_O.

In order to validate our findings, we compared regional lung ventilation and perfusion from EIT using hypertonic saline (NaCl 10%, 10 mL) with SPECT at two different PEEP levels (0 and 15 cmH_2_O) [65]. While there has been a sufficient correlation between EIT and SPECT for both ventilation and perfusion, we observed a spatial mismatch between ventilation and perfusion which might spoil the pixel-wise calculation of regional V/Q. These findings need to be further investigated as the origin of the spatial mismatch can be attributed to several factors: EIT reconstruction, errors in the gamma-variate model assumption, and the ambiguity arising from the 3D volume projection of the EIT sensitivity region to the 2D image plane.

### 6.2. Perfusion Monitoring Using Cardiac Activity

#### 6.2.1. Measurement Principle

In the EIT waveform, cardiac activity (CRS) is observed in the heart as well as in the lung region [72]. Impedance changes in both regions can mainly be attributed to localized changes in blood volume. As blood has a higher conductivity (and thus a lower impedance) than tissue, an impedance decrease or increase can be observed when blood enters or leaves a region, respectively. In the heart region, an impedance increase is observed during systole, whereas an impedance decreases results during diastole. In the lung region, contradictory and delayed behavior is observed. During systole a decrease of impedance results, whereas an increase is observed during diastole [73]. The impedance changes are likely to due to volume changes resulting from the arterial pulse-wave traveling from the heart into the pulmonary vascular tree. The exact origin and composition of the impedance changes are incompletely understood and can only partly be attributed to lung perfusion [13]. Most probably, the flow-speed of blood, as well as mechanic deformation and movement of organs, also causes alterations in regional impedance.

#### 6.2.2. Separating Ventilation and Cardiac-Related Signals

In order to enable real-time analysis of regional perfusion, techniques to separate cardiac- and ventilation-related EIT waveforms are of great importance. A major difficulty is the difference in amplitude of the two signals, because the CRS is an order of magnitude smaller than the VRS [74]. Several methods exist, including short-time apnea, ECG gating, frequency filtering as well as more advanced signal processing techniques. While setting the mechanical ventilator on short-time apnea is not an actual separation technique, it is a very efficient way to obtain a clear recording of the CRS. Repeated periods of apnea might be problematic in some patients. For an ECG gating [75,76], a synchronously recorded ECG is used as a trigger to average a high number (e.g., 200) of heart cycles from the EIT waveform. Assuming that VRS and CRS are uncorrelated, the CRS will be amplified while the VRS is suppressed. A very good separation result, but EIT waveforms are delayed due to the averaging process, which prevents real-time analysis. Frequency filtering [77,78] assumes, that the frequency spectra of VRS and CRS are disjoint uses digital filters to obtain the CRS from the mixed EIT waveform in real-time. Problems arise with high breathing or low heart rates because of spectral overlap. Promising results have been achieved with more advanced signal processing techniques. Specifically, principal component analysis (PCA) has been proven to be useful for extracting cardiac template functions from combined EIT waveforms [79]. Linear fitting of these template functions enables efficient, real-time separation of VRS and CRS.

#### 6.2.3. Validation Studies

Regional perfusion based on the CRS has been compared to radionuclide scintigraphy [80], magnetic resonance imaging (MRI) [72], and single-photon emission computed tomography (SPECT) [64]. Further investigations of pulmonary perfusion using the CRS involved modification of pulmonary perfusion using a balloon catheter [81], OLV [77], and repositioning of EIT belt and patient [73] are needed. Stroke volume estimation using the CRS has been explored in animal [82] and human [76,83] studies. Recently, interesting simulative studies have shown limitations and challenges [84] as well as the influence of heart motion [85] on CRS-based stroke volume estimation.

## 7. Conclusions

EIT creates noninvasive and radiation-free images of the regional ventilation and lung perfusion distribution at the bedside. Functional EIT images allow different functional characteristics of the respiratory and cardio-circulatory system to be addressed. EIT can be combined with other signals, such as airway pressure, enabling the assessment of regional respiratory system compliance (C_RS_) or regional pressure/volume (P/V) curves. Changes in lung ventilation and EELV, compliance, CRS, P/V curves, as well as alveolar overdistension and collapse can be assessed by EIT dynamically on a regional level. The high temporal resolution of EIT allows the analysis of aeration and ventilation time courses to determine the temporal ventilation heterogeneity and may help guide mechanical ventilation in ARDS. Recently, EIT has been shown useful for monitoring of regional lung perfusion as well as stroke volume estimation. Continuous and noninvasive perfusion monitoring can be performed based on the EIT waveforms resulting from cardiac activity in both the heart and lung region, whereas intermittent and invasive monitoring is possible using contrast-agents such as hypertonic saline injected through a central venous line. Monitoring of regional lung perfusion and V/Q imaging using EIT might become available in ARDS patients at bedside.

## Figures and Tables

**Table 1:** Electrical resistivity of thoracic tissues.

Tissue	Resistivity (Ω·cm)
Blood	150
Lungs, inspiration	2400
Lungs, expiration	700
Heart muscle, longitudinal	125
Heart muscle, transversal	1800
Skeletal muscle, longitudinal	160–575
Skeletal muscle, transversal	420–5200
Fat	2000–2700
Bone	16,600

**Table 2:** Commercially available electrical impedance tomography (EIT) devices.

Manufacturer	EIT System	Electrodes		Image Reconstruction Algorithm	Measurement and Data Acquisition
		Number	Configuration		
Swisstom AG	BB^2^	32	electrode belt	Graz consensus reconstruction algorithm for EIT (GREIT)	pair drive (adjustable skip)
				algorithm for EIT (GREIT)	serial measurement
Timpel SA	Enlight	32	electrode stripes	Finite Element Method-based Newton-Raphson method	pair drive (3-electrode skip)
					parallel measurement
CareFusion	Goe-MF II	16	individual electrodes	Sheffield back-projection	pair drive (adjacent)
					serial measurement
Dräger Medical	PulmoVista 500	16	electrode belt	Finite Element Method-based Newton-Raphson method	pair drive (adjacent)
					serial measurement
Maltron Inc	Mark 1	16	individual electrodes	Sheffield back-projection	pair drive (adjacent)
	Mark 3.5	8	individual electrodes		serial measurement
